# Living yeast-based biostimulants: different genes for the same results?

**DOI:** 10.3389/fpls.2023.1171564

**Published:** 2023-06-19

**Authors:** Marie Chambard, Benjamin Albert, Mickaël Cadiou, Sarah Auby, Camille Profizi, Isabelle Boulogne

**Affiliations:** ^1^ Univ Rouen Normandie, GLYCOMEV UR 4358, SFR Normandie Végétal FED 4277, Innovation Chimie Carnot, IRIB, Rouen, France; ^2^ Agrauxine by Lesaffre, Beaucouzé, France

**Keywords:** saccharomyces cerevisiae, soybean, seed coating, reproducibility, RNAseq, abiotic stress tolerance, cell wall and carbohydrate synthesis

## Abstract

Nowadays, many products are available in the plant biostimulants market. Among them, living yeast-based biostimulants are also commercialized. Given the living aspect of these last products, the reproducibility of their effects should be investigated to ensure end-users’ confidence. Therefore, this study aimed to compare the effects of a living yeast-based biostimulant between two different soybean cultures. These two cultures named C1 and C2 were conducted on the same variety and soil but in different locations and dates until the VC developmental stage (unifoliate leaves unrolled), with *Bradyrhizobium japonicum* (control and Bs condition) and with and without biostimulant coating seed treatment. The foliar transcriptomic analysis done first showed a high gene expression difference between the two cultures. Despite this first result, a secondary analysis seemed to show that this biostimulant led to a similar pathway enhancement in plants and with common genes even if the expressed genes were different between the two cultures. The pathways which seem to be reproducibly impacted by this living yeast-based biostimulant are abiotic stress tolerance and cell wall/carbohydrate synthesis. Impacting these pathways may protect the plant from abiotic stresses and maintain a higher level of sugars in plant.

## 1 Introduction

The fact that phytosanitary products are widely criticized and the need to increase productivity while helping plants to grow in adverse environments has recently brought plant biostimulants into the equation (du Jardin et al., 2020). Biostimulants are natural-based solutions that adhere to the principles of organic farming; they are assumed to have a low impact on the environment or human health and thus could contribute to reducing the use of chemical products (La Torre et al., 2016; du Jardin et al., 2020). Referring to the European regulation (EU) 2019/1009, a biostimulant is a substance, a mixture, or a microorganism applied on plants or rhizosphere acting independently from its nutrient content that stimulates nutrition efficiency, soil nutrients availability, products quality, or abiotic stress tolerance (European Commission, 2019). Referring now to scientific literature, a consensus agrees to define biostimulants around five main categories: humic substances, amino acids and protein derivatives, non-nutritive inorganic molecules, land plant and algal extracts, and microbial substances including beneficial bacteria, filamentous fungi, or yeasts (du Jardin, 2015; Van Oosten et al., 2017; Yakhin et al., 2017).

Within a few years, interest in biostimulants and related literature has quadrupled (du Jardin et al., 2020). However, microbial biostimulants and especially yeast-based products seem to be less studied than others. Nevertheless, yeast-based plant biostimulant effects were observed on yield and abiotic stress tolerance of various plants such as wild rocket (*Diplotaxis tenuifolia* L.) (Schiattone et al., 2021), tomato (*Solanum lycopersicum* L.) (Lonhienne et al., 2014; Mannino et al., 2020; Campobenedetto et al., 2021), rice (*Oryza sativa* L.) (Johnson and Puthur, 2022), flax (*Linum usitatissimum* L.) (Emam, 2013), chia (*Salvia hispanica* L.) (Esmail et al., 2022), maize (*Zea mays* L.) (Abdelaal et al., 2017), navel orange (*Citrus sinensis* L.) (El-Boray et al., 2015), and sugar cane (*Saccharum officinarum* L.) (Lonhienne et al., 2014).

In these studies, yeast extracts were sometimes used in a mixture with seaweed extract. Indeed, in wild rockets (*Diplotaxis tenuifolia* L.), a fluid extract from brown algae and yeast applied on leaves increased leaf chlorophyll content and decreased antioxidant activity. With the foliar application of another mixture, an increase in the antioxidant contents of tomato (*Solanum lycopersicum* L.) fruits (Mannino et al., 2020) as well as fruit yield, size, and nutritional composition, and a decrease in fruit ripening time was demonstrated. Another such formulation was also investigated during drought stress in tomato (*Solanum lycopersicum* L.), and it seemed to mitigate this stress (Campobenedetto et al., 2021).

Some of these studies use only yeast and especially *Saccharomyces cerevisiae* in extract form. Seeds priming of rice (*Oryza sativa* L.) with a yeast extract from *S. cerevisiae* biostimulant maintained cell homeostasis and provided a better adaptation to nutrient deficiency stress (Johnson and Puthur, 2022). An *S. cerevisiae* yeast extract combined with a phytohormone-based biostimulant was applied at the bloom stage and one month later was able to induce an increase in yield, fruit set, and fruit quality in navel orange (El-Boray et al., 2015). Another study highlighted that a yeast extract biostimulant applied three times on soil starting 45 days after sowing was not as effective as fertilizers but was still able to significantly increase the quality component (fatty acid content) of chia (*Salvia hispanica* L.) (Abdelaal et al., 2017).

Lastly, very few of them deal with the living-based formulation. It was the case in a study where an active dry *S. cerevisiae* yeast biostimulant applied during germination in salt stress condition, was shown to improve flax (*Linum usitatissimum* L.) seedling growth and restore membrane integrity (Emam, 2013). Living *S. cerevisiae* yeast applied in soil was shown to increase root and shoot biomass and nitrogen and phosphorus content in tomato and sugarcane plants (*Saccharum officinarum* L.) (Lonhienne et al., 2014). Another such treatment applied in irrigation water 30 and 45 days after sowing led to significant increases in leaf biomass (number and area), chlorophyll concentration, relative water content, and antioxidant enzymes activity in maize (*Zea mays* L.) plants in drought stress conditions (Abdelaal et al., 2017).

Thus, despite the low amount of studies on yeast-based plant biostimulants, and the even lower amount of studies on living yeast-based biostimulants, yeast seems to be an efficient solution for more sustainable agriculture (Hernández-Fernández et al., 2021). However, the living yeast biostimulant effect may be modulated according to environmental and cultural parameters. Putative effect modulation could also be due to possible changes in the soil microorganism’s community as the biostimulant is composed of living organisms. Due to the low number of studies on living yeast-based biostimulants, the reproducibility of their effects according to the soil type or environmental conditions still needs to be investigated. To test this reproducibility, we aimed to compare the effect of a living yeast-based plant biostimulant in two slightly different experiments, recreating differences between two replicates of a culture in the same field plot, as farmers would do in their fields from year to year. In this study, the chosen plant model was soybean (*Glycine max* (L.) Merr.), which is a highly and worldwide cultivated plant thanks to its seeds that are mainly used in human and animal nutrition (Kasai, 2017). Soybean seeds were coated with the nodulating bacteria *Bradyrhizobium japonicum* as it would be done in the field, and a living yeast-based biostimulant was applied on seeds before sowing, as recommended by the biostimulant manufacturer. To the best of our knowledge, no study was conducted on this plant model, with the seed-coating treatment of yeast-based biostimulant combined with a high throughput transcriptomic analysis. Two soybean cultures were therefore conducted in greenhouses, using the same variety and seed treatment and the same loam soil. These two cultures were done on different dates and geographical emplacements, reproducing differences between the two crop seasons. A transcriptomic analysis was performed on soybean leaves at the VC stage, allowing a fine observation at the transcripts level of the living yeast-based biostimulant effect between these two cultures.

## 2 Results and discussion

In this study, the effect of a living *Saccharomyces cerevisiae*-based biostimulant (Bs) was investigated in two different soybean cultures using transcriptomic analysis. The first culture, named C1, was conducted in March in Normandy (France), and the second one located 300 km from C1, named C2, was conducted in October in Pays de la Loire (France). The seed batch, soil, and greenhouse settings were the same for these two cultures. Between C1 and C2, the difference was the location and growing period, which led to sunlight differences. Indeed, stronger sunlight was observed during culture C1 in Normandy (data not shown).

### 2.1 Global analysis

**Figure 1:**
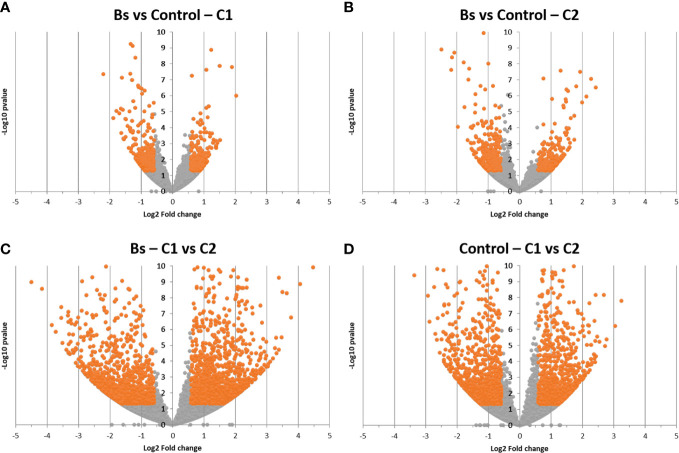
Volcanoplot analysis of differentially expressed genes upon biostimulant treatment for the two cultures **(A, B)** or between the two cultures for the same condition **(C, D)**. Genes are represented by the points; the orange points correspond to significantly differentially expressed genes (fold change (FC) ≤ 0.66 or FC ≥ 1.5 corresponding to Log2(FC) ≤ −0.585 or Log2(FC) ≥ 0.585; p-value ≤ 0.05, corresponding to −Log10(p-value) ≥ 1.30103). Biostimulant (Bs); C1, culture conducted in Normandy, and C2, culture in Pays de la Loire.

Firstly, differential gene expressions were observed between biostimulants treatment (Bs) and control (*Bradyrhizobium japonicum* treated seeds) conditions (**Figures 1A, B**). For the two cultures, we observed differentially expressed genes upon Bs treatment, meaning that soybean plants were responding to the biostimulant treatment on seeds. A comparable amount of differentially expressed genes was found between the two cultures. Indeed, for the C1 culture, 531 under-expressed and 437 over-expressed genes were found. For the C2 culture, 516 under-expressed and 583 over-expressed genes were found among the 42,007 genes described in soybean (Valliyodan et al., 2019).

To get a similar response to the Bs treatment that was observed in the first analysis, another gene differential expression analysis was performed. This time, the comparison was between the two cultures in the same condition: Bs or control condition (**Figures 1C, D**). In the Bs condition, 2918 genes were differentially expressed between the two cultures (1469 genes were over-expressed in C1 and 1449 genes were over-expressed in C2). In the control condition, 2310 genes were differentially expressed between C1 and C2 (1157 genes were over-expressed in C1 and 1153 genes were over-expressed in C2).

**Figure 2:**
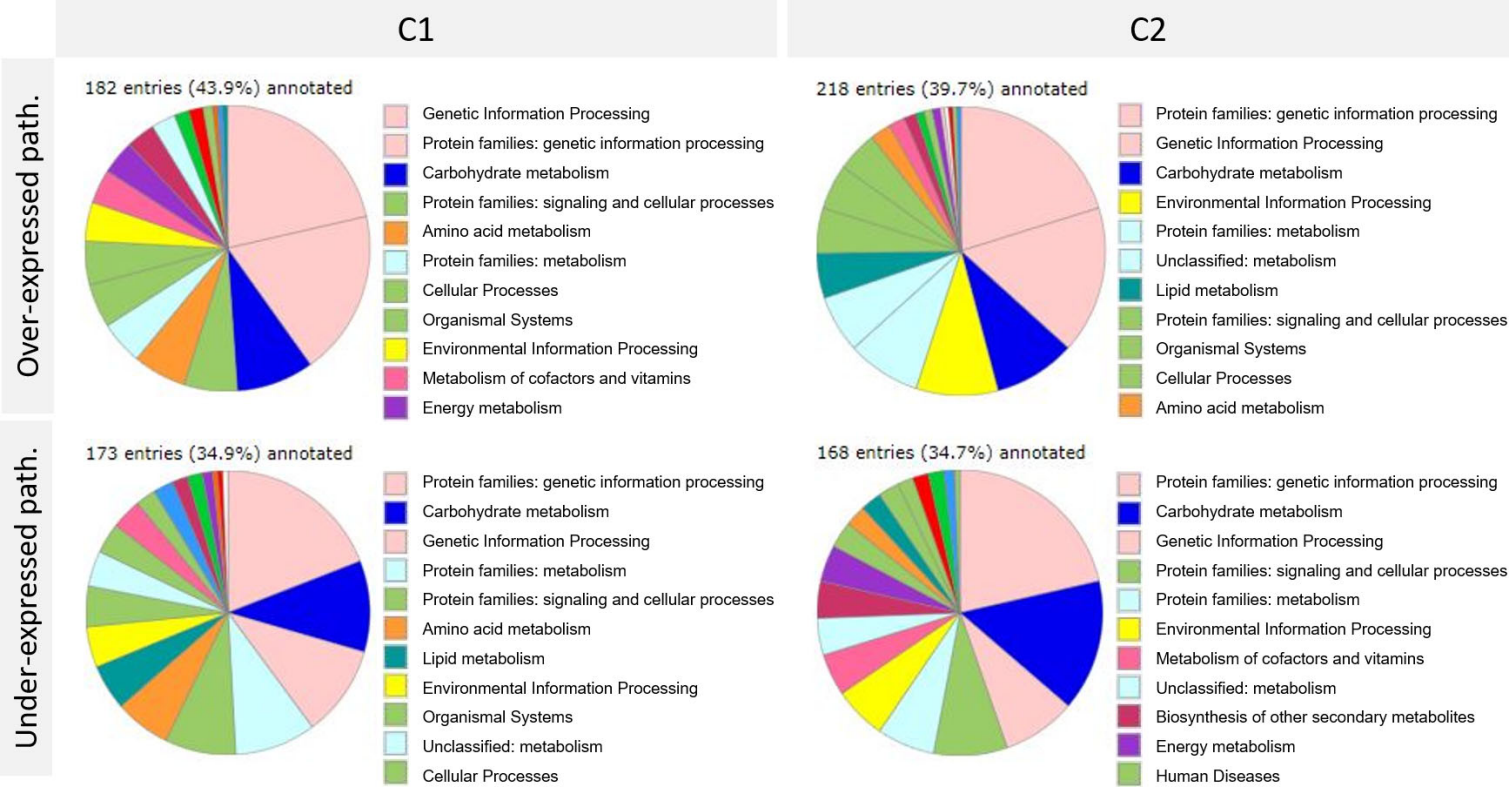
Pathways analysis of differentially expressed genes upon biostimulant (Bs) treatment in C1 and C2 cultures was achieved thanks to BlastKOALA provided by the KEGG database. Path. = pathways. C1, culture conducted in Normandy, and C2, culture in Pays de la Loire.

These two differential analyses showed a clear difference in gene expression between the two cultures C1 and C2 at the same plant development stage and for the same condition (control or Bs). Further analysis was performed, focusing on pathways corresponding to the differentially expressed genes upon Bs treatment. One of the tools used for this analysis was BlastKOALA, provided by the KEGG database (**Figure 2**).

Despite the low number of annotated genes (between 34 and 43%), this analysis showed a clear majority involved in pathways such as genetic information processing and carbohydrate synthesis found in C1 and C2 and over and under-expressed genes. Other pathways were also impacted as signaling and cellular processes and metabolism. Pathways involved in the response to Bs treatment seemed to be very similar between cultures C1 and C2.

To further confirm our observation, other analyses were conducted with tools such as Panther DB (Mi et al., 2021), Mapman (Thimm et al., 2004), or WeGO (Ye et al., 2018) (**Tables S1, S2**; **Figures S1, S2**). According to these tools, pathways that seem to be involved in Bs treatment response in soybean cultivated in C1 and C2 are reported in **Table 1**. Our observations highlighted 15 pathways differentially expressed in response to the Bs treatment which were highly found in each analysis. Among these 15 pathways, only one pathway for each culture seemed culture-specifically represented: negative DNA recombination for C1 and catalytic activities regulation for C2. The 14 other pathways were found to be induced in response to the Bs seed treatment in both cultures (C1 and C2). This result was surprising, considering the high number of differentially expressed genes between the two cultures. Indeed, the first hypothesis was that the pathway analysis would have resulted in different pathways impacted by the Bs treatment between the two cultures. Consequently, further analysis of genes involved in these different pathways for each culture was done.

**Table 1:** The summary of observed pathways involved in response to biostimulant (Bs) treatment in two different cultures C1 and C2.

Pathways	C1	C2
Abiotic stress resistance	+/−	+/−
Carbohydrate synthesis	+/−	+
Catalytic activities regulation		+
Cell wall metabolism	+	+
Energy metabolism	+/−	+/−
Genetic information processing	+/−	+/−
Growth and development	+	+/−
Lipids metabolism	+/−	+
Negative regulation of DNA recombination	+	
Nitrogen transport and use	+/−	+/−
Nodulation	+	+
Phytohormones	+/−	+/−
Proteins metabolism	+/−	+/−
Signalization	+/−	+/−
Specialized metabolism	+/−	+/−
Transcription factors	+/−	+/−

Pathways observed thanks to mainly over-expressed genes are annotated with “+” and pathways observed with mainly under-expressed genes are annotated “−”. “+/−” means there were over- and under-expressed genes involved in this pathway. The grey boxes stand for pathways that were not observed to be involved in response to the Bs treatment in C1 or C2. Results were obtained according to analyses performed with tools such as BlastKOALA, Panther DB, Mapman, and WeGO. C1, culture conducted in Normandy, and C2, culture in Pays de la Loire.

### 2.2 Commonly regulated pathways between C1 and C2

Firstly, commonly regulated pathways between C1 and C2 were investigated (**Table 1**). These 14 pathways are ‘abiotic stress resistance’, ‘carbohydrate synthesis’, ‘cell wall metabolism’, ‘energy metabolism’, ‘genetic information processing’, ‘growth and development’, ‘lipids metabolism’, ‘nitrogen transport and use’, ‘nodulation’, ‘phytohormones’, ‘proteins metabolism’, ‘signalization’, ‘specialized metabolism’, and ‘transcription factors’.

The pathway abiotic stress resistance in culture C1 was mainly highlighted thanks to genes corresponding to cell wall components and carbohydrate synthesis genes, transcription factors, kinases, amino acids metabolism, specialized metabolism genes, heat shock proteins, and a germin-like coding gene. In culture C2, genes involved in abiotic stress resistance corresponded to cell wall components and carbohydrate synthesis genes, transcription factors, kinases, peroxidases, lipid metabolism genes, G proteins, and TPR (tetratrico peptide repeats) domain-containing proteins.

In C1, among genes involved in cell wall components, over-expressed genes such as cellulose synthase, pectin-methylesterases (PME), and galacturonosyltransferase involved in pectins synthesis (cell wall components) were found. Cellulose and pectin are important components of the cell wall (Anderson and Kieber, 2020). The cell wall has many biological and physical functions of which abiotic stress tolerance such as drought (Leucci et al., 2008) or salt (An et al., 2014) stresses can be cited. PME are cell wall remodeling enzymes allowing the formation of a strengthening pectin gel (Micheli, 2001) in optimal conditions and upon stress conditions (Pelloux et al., 2007; Wu et al., 2018). In C2, genes involved in carbohydrate synthesis and cell wall metabolism were also found. Concerning cell wall, genes were PME, xyloglucan endotransglycosydases (XTH), precursors synthesis, and enzymes degrading mannans (hexokinase and mannosidase). XTH are enzymes responsible for xyloglucan polymer modification and the formation of xyloglucan and cellulose cross-linking, and regulating cell wall extensibility (Song et al., 2018).

For carbohydrate synthesis in C1, a raffinose synthase coding gene was also detected. Raffinose is known as an osmoprotectant involved in water stress response (Nishizawa et al., 2008). Genes involved in glycolysis and starch synthesis were also found as part of the carbohydrate synthesis pathway in C1 in response to Bs treatment. In C2, trehalose synthesis genes were detected among carbohydrate synthesis genes. This disaccharide plays a role in growth and development control and stress resistance (Phan and Van Dijck, 2019).

Genes involved in energy metabolism, especially in glycolysis (enolases and hexokinases) and starch synthesis, were also found in C2 as part of the carbohydrate synthesis pathway represented.

The genetic information processing pathway was represented by genes involved in RNA splicing with genes coding for spliceosome subunits (Wilkinson et al., 2020) or in protein synthesis with genes coding for ribosomal subunits (Martinez-Seidel et al., 2020).

The pathway of growth and development was found in C1 thanks to various genes such as G-proteins coding genes (Ofoe, 2021), a germin-like protein coding gene (Dunwell et al., 2008), or genes involved in auxin metabolism (Teale et al., 2006). In C2, differentially expressed genes coding for G-proteins were also detected, as well as genes involved in auxin and abscisic acid (Chen et al., 2020) metabolism.

Some differentially expressed genes involved in lipid metabolism were detected in C1 and C2 as over-expressed phospholipase genes and under-expressed ligase coding genes involved in a lipid precursor synthesis in C1. In C2, synthase coding genes involved in lipids synthesis and elongation and a diacylglycerol kinase coding gene involved in membrane sphingolipid synthesis were over-expressed. These lipids participate in the development, stimulus sensing, and stress response (Liu et al., 2021). Two ligases involved in lipid elongation and two lipid degrading kinases were under-expressed.

Nodulation processes seemed impacted by the Bs seed treatment, notably with the over-expression of transcription factors involved in nodulation such as bZIP (basic leucine zipper), MYB (myeloblastosis), and bHLH transcription factors (Du et al., 2012; Chiasson et al., 2014; Wang et al., 2015). MYB transcription factors functions are highly diverse but appear to play an important role during nodulation in soybean (Du et al., 2012), as well as bZIP transcription factors which are involved in plant development, including nodulation processes (Wang et al., 2015). bHLH transcription factors are involved in nodules growth and ammonium transport in soybean (Chiasson et al., 2014).

The phytohormones pathway was mostly represented in both cultures by under-expressed genes from ethylene and auxin metabolism. In soybean, ethylene is known to be involved in flowering and fruit development and the negative regulation of abiotic stresses such as cold stress (Robison et al., 2019). Auxin is known to be involved in growth regulation (Teale et al., 2006).

The protein metabolism pathway was largely impacted in the two cultures C1 and C2, for instance, with the differential expression of many ribosomal proteins involved in protein synthesis (Martinez-Seidel et al., 2020) and MAP kinases (mitogen-activated protein kinases) involved in protein phosphorylation (Kumar et al., 2020).

The signalization pathway involved kinases receptors LRR-RLK (leucine-rich repeat receptor-like kinase) in C1 and C2. More specifically, LRR-XI and LRR-III family RLK were detected. LRR-XI RLKs are known to play a role in growth and development (size control of the apical meristem, floral meristem, and floral organ abscission) in response to nodulation factors and during water stress (Meng et al., 2020; Soltabayeva et al., 2022). LRR-III RLKs have major functions in secondary cell wall formation, silique formation, and organ and tissue development (Meng et al., 2020).

The specialized metabolism also seemed impacted in C1 and C2, especially with under-expressed genes involved in flavonoids metabolism as chalcone synthase genes playing a role in naringenin synthesis, which is involved in tolerance to salt and osmotic stress (Ozfidan-Konakci et al., 2020).

Many transcription factors from different pathways were also detected in C1 and C2, among these, bZIP (basic leucine zipper) and MYB (myeloblastosis) transcription factors. The differential expression of these transcription factors mainly explained the appearance of the nodulation process pathway among the commonly regulated pathways. In C1 and C2, bHLH transcription factors were also found. These transcription factors are involved in nodules growth and ammonium transport in soybean (Chiasson et al., 2014), as well as abiotic stress response (Li et al., 2018).

With the analysis of these common pathways differentially expressed upon Bs treatment in the two cultures C1 and C2, we found four categories of pathways that have redundant genes: abiotic stress resistance, cell wall and carbohydrate synthesis, growth and development, and signalization.

### 2.3 Commonly regulated genes between C1 and C2

Lastly, an analysis focusing only on common genes differentially expressed in both cultures was conducted. This analysis highlighted 18 over-expressed (**Table 2**) and 60 under-expressed genes (**Table S3**).

**Table 2:** Commonly over-expressed genes between C1 and C2. A blast against all plants’ genomes was done with uncharacterized proteins.

Phytozome ID	Protein names/BLAST	Pathways
GLYMA_01G037900	Hexosyltransferase (EC 2.4.1.-)	Carbohydrate synthesis
GLYMA_01G155600	Protein kinase domain-containing protein	Signaling
GLYMA_02G039700	Classical arabinogalactan protein 5-like	Carbohydrate synthesis, growth, programmed cell death, communication with microorganisms, and stress tolerance
GLYMA_05G042000	Uncharacterized protein/Protein phosphatase 2c, putative (Medicago truncatula; 84,5% identity)	Abiotic stress
GLYMA_05G131200	CAF1C_H4-bd domain-containing protein	Histone chaperone
GLYMA_06G093600	Peptidase A1 domain-containing protein	Protein degradation, cell death, and stress response
GLYMA_06G246500	Pentatricopeptide repeat-containing protein	Abiotic stress, RNA metabolism, and ROS generation
GLYMA_07G051500	BHLH transcription factor	Abiotic stress, nodulation, and ammonium transport
GLYMA_09G022000	Patatin (EC 3.1.1.-)	Carbohydrate synthesis
GLYMA_10G179200	Protein EXORDIUM-like 5	Abiotic stress
GLYMA_11G117600	CIRBP cold-inducible RNA-binding protein	Abiotic stress
GLYMA_13G145000	Phenylalanine ammonia-lyase (EC 4.3.1.24)	Abiotic stress and secondary cell wall metabolism
GLYMA_13G206700	TPT domain-containing protein	Transport
GLYMA_13G262800	FBD domain-containing protein	Protein degradation, cell death, stress response
GLYMA_16G023500	Hydroxyproline-rich glycoprotein family protein	Abiotic stress and growth, development
GLYMA_17G087500	SOUL heme-binding protein	Electron transport, oxygen carrier, and enzyme cofactor
GLYMA_17G176200	3-ketodihydrosphingosine reductase	Lipid metabolism
GLYMA_19G043000	Proline dehydrogenase (EC 1.5.5.2)	Proline and arginine metabolism

C1, culture conducted in Normandy, and C2, culture conducted in Pays de la Loire.

Among the 18 over-expressed genes in C1 and C2 with Bs treatment, seven genes were involved in abiotic stress tolerance mechanisms. A phenylalanine ammonia-lyase coding gene (GLYMA_13G145000) involved in monolignol and naringenin synthesis was over-expressed. These two previous compounds are, respectively, involved in secondary cell wall rigidity and hydrophobicity (Soltabayeva et al., 2022), and tolerance to salt and osmotic stress (Ozfidan-Konakci et al., 2020). An HRGP (hydroxyproline-rich glycoprotein) coding gene (GLYMA_16G023500) was also found. HRGPs are cell wall glycoproteins involved in abiotic stress response but also growth and development pathways (Kieliszewski and Shpak, 2001; Deepak et al., 2010). Another gene that seems to play a role in abiotic stress tolerance is a cold-inducible RNA-binding protein (CIRBP) coding gene (GLYMA_11G117600) known for being induced in various stresses including cold stress (Sachetto-Martins et al., 2000; Brochu et al., 2013). A basic helix-loop-helix (bHLH) transcription factor (GLYMA_07G051500) involved in abiotic stress response (iron deficiency), nodulation, and ammonium transport (Chiasson et al., 2014; Cheng et al., 2018) was also detected among over-expressed genes in C1 and C2. A protein phosphatase (GLYMA_05G042000, *Medicago trunculata* 84.5% identity) involved in stress signaling responses during nutrient deficiency and in growth and development (Bheri et al., 2021), a pentatricopeptide repeat-containing protein (GLYMA_06G246500) involved in RNA metabolism (translation, splicing, and editing), ROS (Reactive Oxygen Species) generation, and abiotic stress resistance (Xu et al., 2018), and an EXORDIUM-like 5 protein (GLYMA_10G179200) involved in abiotic stress response such as low carbon availability, sugar starvation, prolonged darkness, and anoxia (Schröder et al., 2011; Hansch et al., 2020) were also over-expressed in the two cultures.

Four genes involved in cell wall/carbohydrate synthesis pathway were also commonly over-expressed in C1 and C2 upon Bs treatment: an HRGP (GLYMA_16G023500), a hexosyltransferase (GLYMA_01G037900) involved in pectin synthesis, a cell wall polysaccharide (Yang and Anderson, 2020), an arabinogalactan protein-like (GLYMA_02G039700), a cell wall protein involved in growth, programmed cell death, communication with microorganisms, and stress protection (Nguema-Ona et al., 2013; Nguema-Ona et al., 2014), and a patatin (GLYMA_09G022000), which is a storage glycoprotein (de Souza Cândido et al., 2011). The other eight genes involved in different pathways (signaling, lipid and protein metabolism, *etc.*) which have been detected are described in **Table 2**.

Conversely, the 60 under-expressed genes (**Table S1**) play, in particular, a role in glycolysis. Indeed, it has highlighted two pyruvate kinases (GLYMA_20G136200 and GLYMA_10G255100) which convert phosphoenolpyruvate in pyruvate, a phosphofructokinase (GLYMA_01G005400) which converts β-D-fructose-6P in β-D-fructose-1,6P_2_, and a ligase (GLYMA_07G242100) which converts acetate in acetyl-CoA used to produce citrate from oxaloacetate in citrate cycle. An isocitrate dehydrogenase (GLYMA_19G005100), which synthesizes oxoglutarate from oxaloacetate in the citrate cycle was also under-expressed.

The protein degradation pathway also seemed under-regulated upon Bs treatment. Indeed, a RING-type domain-containing protein (GLYMA_19G037800), corresponding to a ubiquitin ligase and F-box domain-containing genes (GLYMA_08G158200 and GLYMA_13G360600) were under-expressed. F-box proteins have multiple functions, including protein degradation, cell death, or stress response (Kipreos and Pagano, 2000; van den Burg et al., 2008). Other under-expressed genes were also involved in protein conformation and modification, such as an isomerase (GLYMA_03G198200) acting in protein conformation processes, a glutamine-specific methyltransferase (GLYMA_03G027700) regulating protein translation termination (Heurgué-Hamard et al., 2002), and an N-acetylglucosaminyltransferase (GLYMA_05G175000) acting in N-linked glycoproteins synthesis.

Many other genes involved in various processes were found, such as DELLA proteins (GLYMA_03G219900) which are negative regulators of the gibberellic acid pathway (Locascio et al., 2013), a basic leucine zipper (bZIP) transcription factor (GLYMA_03G247100) involved in stress signaling and development (Dröge-Laser et al., 2018), and two zinc finger proteins (GLYMA_04G044900 and GLYMA_05G094700). Zinc finger proteins are involved in many biological processes, including DNA recognition, RNA packaging, transcriptional activation, and apoptosis regulation (Laity et al., 2001). ZAT10 (GLYMA_04G044900) proteins are specifically known to act as a negative regulator of the abiotic stress response (Ciftci-Yilmaz and Mittler, 2008). A gene coding for an ABC (ATP binding cassette) transporter (GLYMA_06G154500) was also found. These transporters are involved in flavonoid transport, detoxification, growth and development, and stress response (Kang et al., 2011; Zhao, 2015). Another gene under-expressed upon Bs treatment that was also found was a gene coding for an EF-hand domain-containing protein (GLYMA_07G053300) involved in Ca^2+^ signaling (Mohanta et al., 2019).

**Figure 3:**
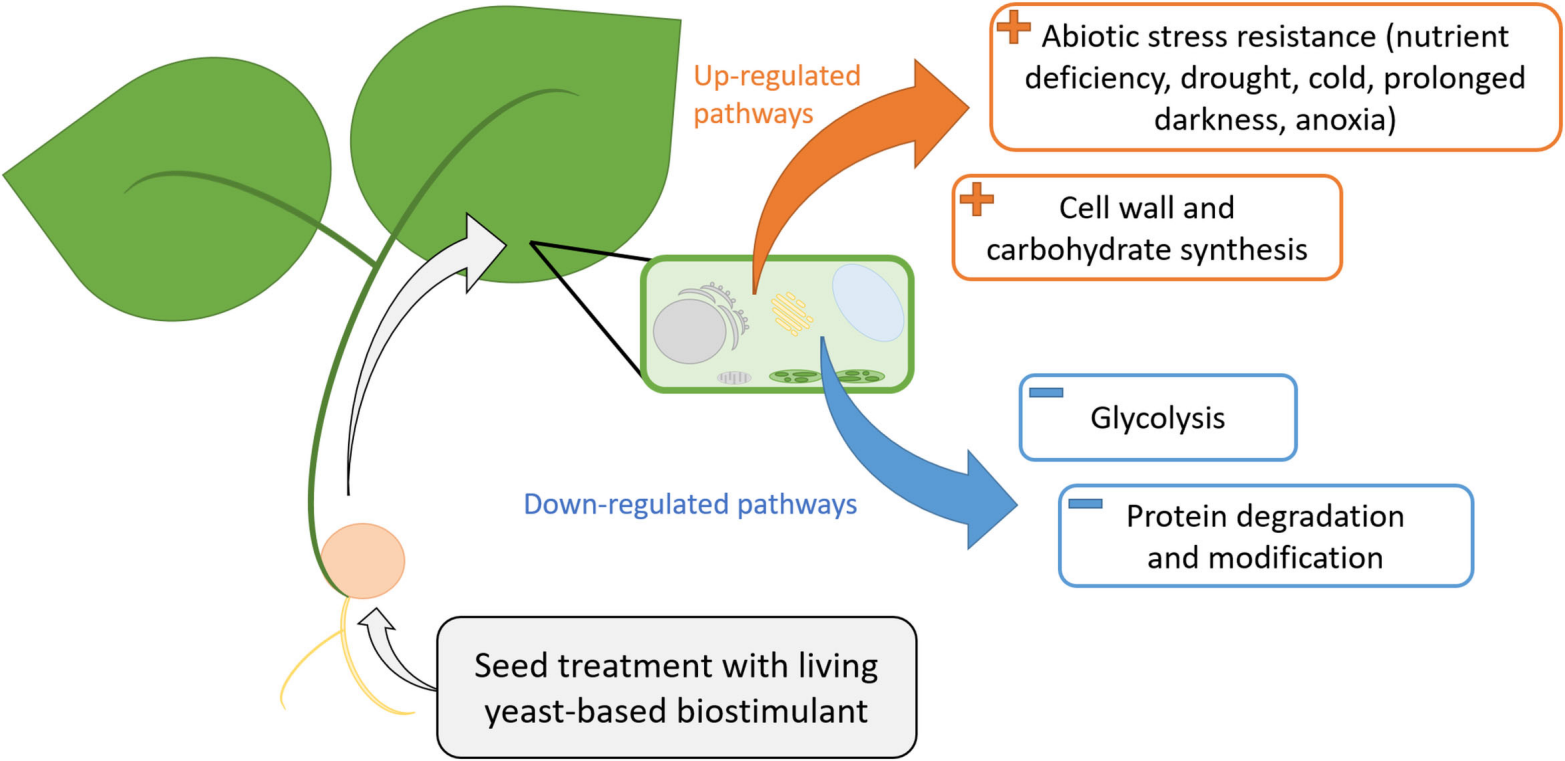
The mode of action of the living *Saccharomyces cerevisiae*-based biostimulant according to commonly up-regulated (orange) and down-regulated (blue) genes in C1 and C2 cultures. Up-regulated genes in C1 and C2 seem to mainly impact abiotic stress resistance pathways and cell wall and carbohydrate synthesis pathways. Down-regulated genes in C1 and C2 seem to mainly impact protein degradation and modification as well as glycolysis pathways. C1, culture conducted in Normandy, and C2, culture in Pays de la Loire.

With this analysis of common genes between both cultures, abiotic stress resistance and cell wall/carbohydrate synthesis seemed to be the two main pathways represented by over-expressed genes in response to Bs seed treatment. Protein degradation, modification, and conformation processes, as well as glycolysis, seem to be the main pathways modified by under-expressed genes (**Figure 3**). The over-expression of genes involved in these pathways and notably abiotic stress resistance pathway seems consistent with previous studies on living-based biostimulants, which were shown to improve flax seedlings’ growth in salt stress conditions (Emam, 2013) and maize resistance to drought stress (Abdelaal et al., 2017).

Further investigation is needed to characterize the molecular interactions leading to these soybean pathway modifications. Such interactions might occur directly between the living yeast-based biostimulant and soybean and between the biostimulant and the soil microbial communities, including *Bradyrhizobium japonicum*. Indeed, a possible interaction between *S. cerevisiae* and *B. japonicum* was shown recently (Zveushe et al., 2023), resulting in an increase in soil inorganic phosphate solubilization and auxin production by yeast. A similar process might have occurred in this experiment. Auxin is known to be involved in many biological processes in plants, such as in signaling, transport, and root development (Teale et al., 2006; Overvoorde et al., 2010), and phosphorus is a highly important nutrient affecting soybean culture yield (Vieira et al., 2023) involved in processes such as nodulation and nitrogen fixation in soybean. The synthesis of these two components by the yeast might improve soybean nutrition and root development which may lead to an up-regulation of abiotic stress resistance and cell wall and carbohydrates synthesis pathways and a down-regulation of glycolysis and protein degradation and modification pathways.

## 3 Conclusion

In the field, the same biostimulant is used in highly different conditions for the same crop, considering parameters such as the location, application timing, weather, and many other parameters which lead to a putative loss or modification of its effect. This study aimed to assess a living *Saccharomyces cerevisiae*-based biostimulant effect between two different cultures, using RNA sequencing by changing two parameters: the sowing date and the culture location. These two parameters also led to differences in sunlight in greenhouses between C1 and C2. As a main observation, despite the high variability of differentially expressed genes between the two cultures, these genes led to the activation or repression of the same pathways, especially abiotic stress resistance, cell wall and carbohydrate synthesis, growth and development, and signalization. Additionally, commonly differentially expressed genes between C1 and C2 upon Bs treatment confirmed that a reproducible effect on genes of this living yeast-based biostimulant may have an impact on abiotic stress resistance, carbohydrate synthesis, protein metabolism, and glycolysis. In conclusion, despite the differences between the two cultures, when merging both analyses, the pathways which seem to be reproducibly impacted by this living yeast-based biostimulant are abiotic stress tolerance and cell wall/carbohydrate synthesis. Thus, the biostimulant may induce these pathways to protect the plant against various abiotic stresses during plant development and maintain a higher level of sugars.

## 4 Materials and methods

### 4.1 Plant material, seed treatments, and cultures

All *Glycine max* (L.) Merr. seeds variety Nessie PZO (Sem-Partners, Maule, France) were treated with 4g/kg of bacteria *Bradyrhizobium japonicum* (FORCE 48 NPPL, Euralis semences, Lescar, France), and for the treated condition (abovementioned Bs), with 1g/kg of a *Saccharomyces cerevisiae* yeast strain (AGX19-009-V, Agrauxine by Lesaffre, Beaucouzé, France). Seed coating was done in a spinning tank developed by Aegilops Application (Val-de-Reuil, France), in collaboration with Aegilops Application (Val-de-Reuil, France).

Two different cultures in greenhouses were done for this study: a first culture in NormanSerre in Mont Saint Aignan (Normandy, France) named C1 in March 2021, and a second one in INEM (Installations Expérimentales Mutualisées) in Beaucouzé (Pays de la Loire, France) named C2 in October 2021. Greenhouses were set at 20°C during the day (14h) and 14°C during the night (10h). In order to avoid high differences in soil microbial communities, the same loam soil from Ryes (Normandy, France) and the same seed batch were used for C1 and C2. Pots were drop by drop watered. Upon the VC stage (12 days after sowing), soybean leaves were collected and crushed in liquid nitrogen. Six replicates per condition were prepared.

### 4.2 RNA extraction and sequencing

RNA extraction was performed as per RNA/DNA Purification Kit instructions (Norgen, Thorold, Canada). RNA quality was assessed with a 2100 Bioanalyzer system (Agilent, Santa Clara, California, USA) and the kit RNA 6000 Pico Kit Quick Start (Agilent, California, USA). For each condition, the four replicates with the best RNA quality were used to create 12 libraries with the Stranded mRNA Prep Ligation kit (Illumina, San Diego, California, USA), and a pre-run on a MiniSeq (mid-output flow cell) and a NovaSeq run (S4 flow cell) were performed on the MGX facility (https://www.mgx.cnrs.fr/).

### 4.3 Bioinformatic analysis

Fastq files were uploaded on the Galaxy platform (Afgan et al., 2016) and trimmed with Trimmomatic v0.36.6 (Bolger et al., 2014); reads were aligned on the *G. max* genome (Gmax Wm82 a4 v1, Phytozome v13) with Hisat2 v2.1.0 (Kim et al., 2015), and gene count was performed with HTSeq-Count v0.9.1 (Anders et al., 2015). Differential expression analysis of genes was conducted with DESeq2 V2 (Love et al., 2014). Volcanoplot were generated from DESeq2 differential expression analysis results (fold change and p-value) on Microsoft Excel. BlastKOALA (https://www.kegg.jp/blastkoala/) was used for KO (KEGG Onthologies) analysis. A BLAST on UniProtKB reference proteomes and Swiss-Prot databases was performed on uncharacterized proteins coded by differentially expressed genes. Only proteins with over 80% identity were considered. Other tools such as WeGO, Mapman, and Panther db were also used in supplemental data (**Tables S1, S2**; **Figures S1, S2**); methods used for these tools are described in supplemental data.

## Data availability statement

The datasets presented in this study can be found in online repositories. The names of the repository/repositories and accession number(s) can be found below: https://www.ebi.ac.uk/ena, PRJEB57693.

## Author contributions

Conceptualization: IB and BA; investigation: MCh and IB; plant culture in Beaucouzé: MCa and SA; writing—original draft preparation: MCh and IB; writing—review and editing: MCh, IB, BA, and CP. All authors have read and agreed to the published version of the manuscript.
