# Supplementary material for: Living yeast-based biostimulants: different genes for the same results?
Source: Front Plant Sci. 2023 Jun 19;14:1171564. doi: 10.3389/fpls.2023.1171564 (PMC10315835; doi:10.3389/fpls.2023.1171564)
Supplement: Supplementary file 1 [file DataSheet_1.docx]

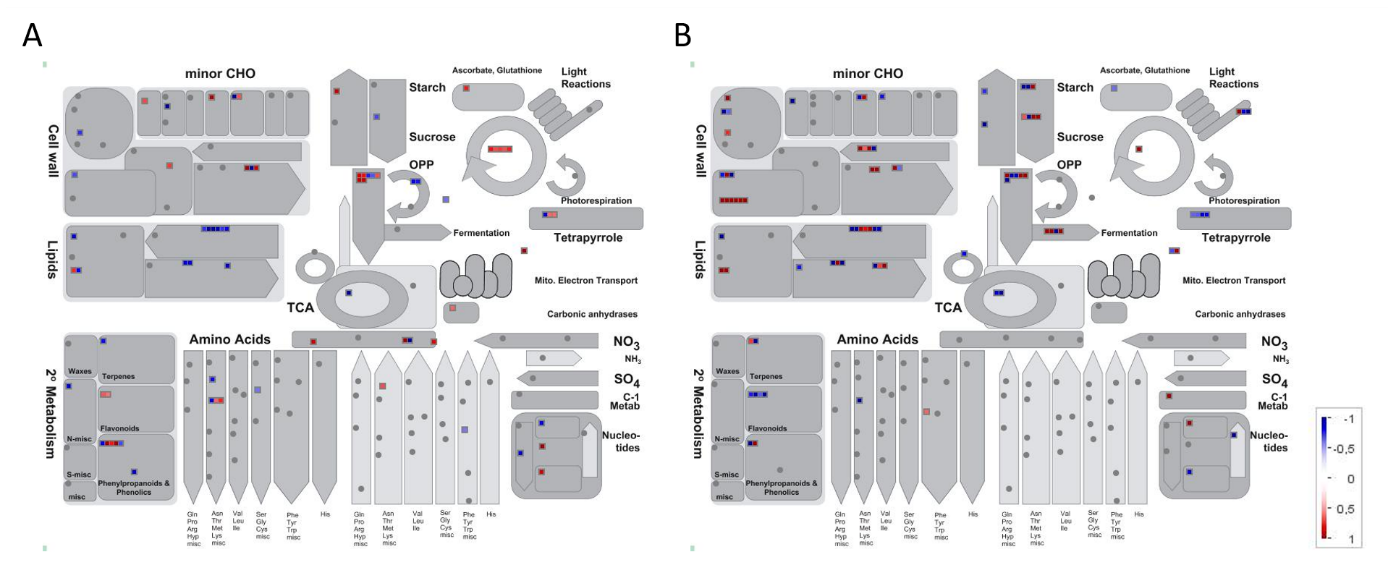
Figure S1. Mapman metabolism overview analysis of differentially expressed genes in C1 (A) and C2 (B) in response to Bs seed treatment. Each differentially expressed gene is represented by a blue or red square. Over-expressed genes are represented in red and under-expressed genes are in blue.


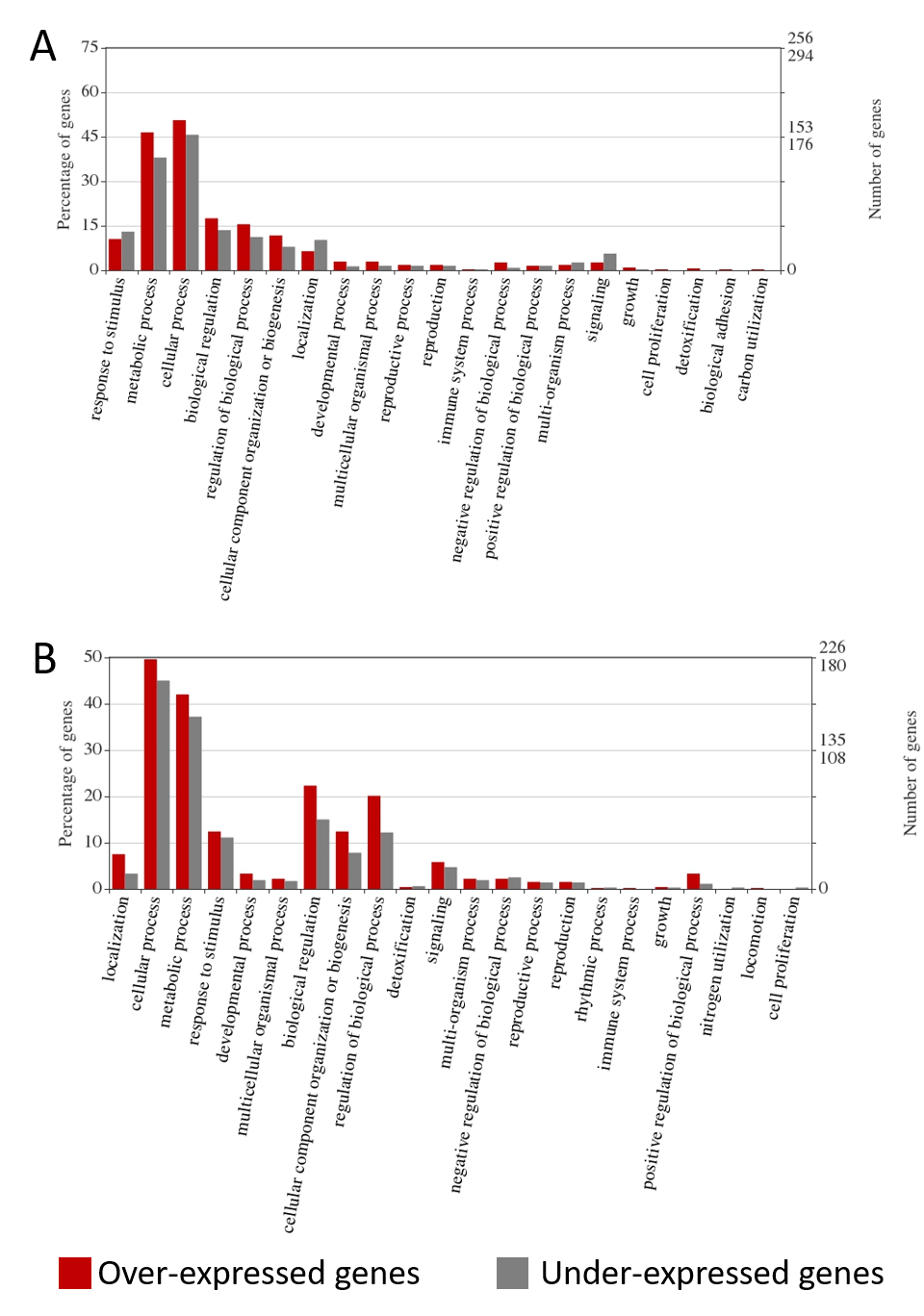


Figure S2. WeGO pathways analysis of differentially expressed genes in C1 (A) and C2 (B) in response to Bs seed treatment.

## Supplementary methods in bioinformatic analysis

A PANTHER Overrepresentation Test (19) was performed on differentially expressed genes in C1 and C2 cultures. The Glycine max reference list was used as a reference and a Fisher’s test with a false discovery rate (FDR) correction was performed. Only genes with a fold enrichment ≥ 2 were considered. Under-expressed gene lists did not result in statistically significant results.

A Mapman metabolism overview analysis (20) was performed on differentially expressed genes in C1 and C2. Gene lists were compared to the Glycine max mapping and the metabolism overview pathway was selected.

A WeGO analysis (21) was performed on differentially expressed genes in C1 and C2. Gene lists were uploaded on WeGO genomics website in native format and the latest gene ontology files (2018-11-01) were selected.
